# Paediatric Non-Alcoholic Fatty Liver Disease: Impact on Patients and Mothers’ Quality of Life

**DOI:** 10.5812/hepatmon.7871

**Published:** 2013-03-12

**Authors:** Luigi Mazzone, Valentina Postorino, Lavinia De Peppo, Claudia Della Corte, Giuseppe Lofino, Lia Vassena, Laura Fatta, Marco Armando, Giorgio Bedogni, Stefano Vicari, Valerio Nobili

**Affiliations:** 1 Department of Neuroscience, Child Neuropsychiatry Unit, IRCCS Children's Hospital Bambino Gesù, Rome, Italy; 2 Department of Dynamic and Clinical Psychology, University of Rome Sapienza, Rome, Italy; 3 Hepato-Metabolic Diseases and Anesthesiology Unit, IRCCS Children's Hospital Bambino Gesù, Rome, Italy

**Keywords:** Depression, Anxiety, Children, Chronic Disease, Adolescence

## Abstract

**Background:**

Non-alcoholic fatty liver disease (NAFLD) is one of the causes of fatty liver in adults and is currently the primary form of chronic liver disease in children and adolescents. However, the psychological outcome (i.e. the behavioural problems that can in turn be related to psychiatric conditions, like anxiety and mood disorders, or lower quality of life) in children and adolescents suffering of NAFLD has not been extensively explored in the literature.

**Objectives:**

The present study aims at evaluating the emotional and behavioural profile in children suffering from NAFLD and the quality of life in their mothers.

**Patients and Methods:**

A total of 57 children (18 females/39 males) with NAFLD were compared to 39 age-matched control children (25 females/14 males). All participants were submitted to the following psychological tools to assess behavior, mood, and anxiety: the Multidimensional Anxiety Scale for Children (MASC), the Child Behavior Checklist (CBCL), and the Children’s Depression Inventory (CDI). Moreover, the mothers of 40 NAFLD and 39 control children completed the World Health Organization Quality of Life-BREF (WHOQOL-BREF) questionnaire.

**Results:**

NAFLD children scored significantly higher as compared to control children in MASC (P = 0.001) and CDI total (P < 0.001) scales. The CBCL also revealed significantly higher scores for NAFLD children in total problems (P = 0.046), internalizing symptoms (P = 0.000) and somatic complaints (P < 0.001). The WHOQOL-BREF revealed significantly lower scores for the mothers of NAFLD children in the overall perception of the quality of life (P < 0.001), and in the “relationships” domain (P = 0.023).

**Conclusions:**

Increased emotional and behavioural problems were detected in children with NAFLD as compared to healthy control children, together with an overall decrease in their mothers’ quality of life. These results support the idea that these patients may benefit from a psychological intervention, ideally involving both children and parents, whose quality of life is likely negatively affected by this disease.

## 1. Background

Non-alcoholic fatty liver disease (NAFLD) was first described by Ludwig in 1980 and has become the primary cause of chronic liver disease in children and adolescents in the last few decades. In Western countries, the incidence of NAFLD is estimated to range from 20 to 30% in adults and from 3 to 10% in children and adolescents (1-4). The acronym NAFLD comprises a broad spectrum of liver conditions. The histological features of NAFLD vary from simple hepatic steatosis (fat accumulation in more than 5% of the hepatocytes) to non-alcoholic steatohepatitis (NASH) with necroinflammation, sometimes associated with fibrosis and eventually progressing to cirrhosis and hepatocellular carcinoma (5). Although children with NAFLD at a cirrhotic stage have been documented, and children with any stage of NAFLD were shown to develop cirrhosis in adulthood (6, 7), the prognosis of paediatric NAFLD with advanced fibrosis or cirrhosis still remains uncertain due to the extremely low number of long-term follow-up studies (8). Aside from adults, children with NAFLD also suffer from metabolic impairments (including increased baseline waist circumference, hypertension and insulin resistance) that increase the risk for type two diabetes mellitus, metabolic syndrome and cardiovascular diseases (9-11). The pathogenic mechanisms leading to NAFLD in both children and adults seem to involve several factors, including genetic background, epigenetics and environmental factors (i.e. high caloric intake, daily consumption of junk food and low level of physical activity, beyond average weight) that all concur during the development and progression of the disease (6-12). Of note, the latest studies estimate a 70–80% incidence of paediatric NAFLD in obese children (4, 13). While there are considerable evidences that has linked obesity to multiple physical health problems (14-16), the relationship between childhood overweight and psychological problems (such as anxiety and mood disorders or lower quality of life) does not seem to be that univocal (17-19). As for NAFLD, to our knowledge, so far only one recent adult study by Elwing et al. in 2006 (20) has investigated the presence of psychiatric conditions in these patients, documenting a higher frequency of anxiety and depression as compared to control subjects, in association with more advanced liver histological abnormalities. However, an extensive evaluation of the psychological outcome including behavioral problems eventually related to psychiatric conditions as well as quality of life in children and adolescents suffering from NAFLD have yet to be done.

## 2. Objectives

In the present study we investigated the emotional and behavioral profile in a sample of children and adolescents suffering from NAFLD with the aim of eventually identifying particular psychological features. Moreover, in order to better characterize the overall psychological picture of pediatric NAFLD, an evaluation of the quality of life in the mothers of the patients was also conducted.

## 3. Patients and Methods

### 3.1. Patients and Controls

A total of 57 children (18 females and 39 males, age range 8 to 17 years), all Caucasians of Italian descent referred to the Hepato-Metabolic Diseases and Anesthesiology Unit of the Children's Hospital Bambino Gesù, of Rome (Italy), and suffering from non-alcoholic fatty liver disease (NAFLD) were included in the study. Thirty-nine age-matched control children (25 females and 14 males) were randomly selected from a database of healthy children attending the pediatric clinic for a normal developmental check-up. Each patient or responsible guardian provided informed consent.

### 3.2. Biochemical Measurements

All patients were tested for alternative causes of steatosis, particularly alcohol abuse (≥ 140 g/week), total parenteral nutrition, and use of medications, e.g. valproate, amiodarone or prednisone, which are known to precipitate steatosis. Appropriate evaluations, based on standard clinical and laboratory analyses and liver biopsies, allowed for the exclusion of concomitant viral infections (Hepatitis A, B, C, D, E, and G viruses, cytomegalovirus and Epstein-Barr), as well as of autoimmune liver disease, metabolic liver disease, Wilson’s disease, and alpha-1-antitrypsin. The study protocol conformed to the ethical guidelines of the Declaration of Helsinki and performed according to the recommendations of the Ethics Committee of our Hospital (Children's Hospital Bambino Gesù of Rome (Italy) (NCT00885313). Informed consent was obtained from each patient or responsible guardian. The body mass index (BMI) (Kg/m2) was calculated accordingly (21). Obesity was defined as BMI ≥ 95th percentile for age and gender (22). The degree of insulin resistance (IR) was determined by the homeostatic model assessment (HOMA-IR) using the formula: IR = (insulin*glucose)/22.5 (23). Serum concentrations of cytokines [Interleukine-6 (normal level serum defined as < 3 pg/ml) and Tumor Necrosis Factor-alpha (normal serum level defined as < 9 pg/ml)] were measured by commercially available kits (Aurogene SRL, 00185, Rome, Italy) based on the Enzyme-Linked Immuno-Sorbant Assay (ELISA). Lipids [total cholesterol (coefficient of variation < 3.0%), triglyceride (coefficient of variation < 3.0%)] and liver enzymes (i.e. aspartate aminotransferase and alanine aminotransferase) were assessed by standard enzymatic methods (10). Table 1 summarizes all the patients included in the study.

**Table 1.** Demographic and Clinical Characteristics of the Sample

	Non-Alcoholic Fatty Liver Disease Group (n = 57)		
	Total (n = 57)	Females (n = 18)	Males (n = 39)
**Age, y, Mean ± SD**	12.07 ± 3.00	11.22 ± 3.11	12.46 ± 2.9
**Age at Onset, y, Mean ± SD**	10 ± 3.00	9.06 ± 2.94	9.92 ± 2.67
**Familiar Psychiatric History, P/A**	14/43	3/13	9/30
**BMI, kg/m^2^, Mean ± SD**	27.05 ± 3.75	27.85 ± 3.76	26.68 ± 3.74
**HOMA-IR, Mean ± SD**	2.53 ± 1.27	3.07 ± 1.52	2.28 ± 1.06
**Liver Enzymes-AST, Mean ± SD**	69.50 ± 57.69	67.83 ± 84.79	70.28 ± 41.07
**Liver Enzymes-ALT, Mean ± SD**	47.00 ± 22.92	43.44 ± 30.08	48.69 ± 18.98
**Cholesterol Levels, Mean ± SD**	159.7 0± 35.37	162.77 ± 28.89	158.38 ± 38.25
**Triglyceride Levels, Mean ± SD**	105.30± 62.08	93.27 ± 37.98	110.84 ± 70.25
**Cytokines-IL-6, Mean± SD**	10.60 ± 7.50	11.32 ± 9.38	10.14 ± 6.21
**Cytokines-TNF-α, Mean ± SD**	22.08 ± 34.50	18.29 ± 16.68	24.11 ± 41.23

Abbreviations: ALT, Alanine Aminotransferase; AST, Aspartate Aminotransferase; BMI, Body Mass Index; HOMA-IR, Homeostasis Model Assessment–Insulin Resistance; IL, Interleukine-6; P/A, Presence/Absence; TNF, Tumor Necrosis Factor-alpha

### 3.3. Liver Biopsy Procedure and Diagnosis of NAFLD

Liver biopsies were performed using an automatic core biopsy device (Biopince, Amedic, Sweden) with an 18-G needle, 150 mm long (10, 24). Biopsy pieces of at least 18 mm were routinely formalin-fixed, paraffin-embedded and stained by Hematoxylin and Eosin and Van Gieson stain for the assessment of fibrosis and architectural changes. They were all examined by a single liver pathologist who was unaware of the patients’ clinical and laboratory data. Diagnosis of NASH performed according to Kleiner and colleagues (24). Steatosis, inflammation (portal and lobular), hepatocyte ballooning and fibrosis were scored according to the Scoring System for Nonalcoholic Fatty Liver Disease developed by the NIH-sponsored NASH Clinical Research Network (CRN) (24).

### 3.4. Assessment of Behavioral and Emotional Problems

All participants were subjected to a battery of psychological tools for a comprehensive evaluation of behavior, depression and anxiety. The Multidimensional Anxiety Scale for Children (MASC), a 39-item four point Likert-style self-reporting scale for children and adolescents was used to measure anxiety symptoms. It has four subscales measuring physical symptoms, social anxiety, harm avoidance and separation anxiety. Raw scores were converted into standard T-scores and a T-score more than 75 indicated the presence of anxiety symptoms. The MASC has been revealed to have good internal consistency (α = 0.60 to α = 0.85) and high test-retest reliability (r = 0.79 to r = 0.93) (25-29). The Italian version of Children’s Depression Inventory (CDI) was used to rate depression symptoms. The CDI is a self-rating scale mostly used to assess depressive symptomatology in children and adolescents aged 8-17 years, with an excellent reliability of 0.87, as measured by Cronbach’s α. This scale consists of 27 items scored on a three-point scale indicating increasing severity of symptoms. According to Italian validation criteria, 19-point cut-off indicates the ideal threshold for a child at risk of depression (30-32). Children and adolescents completed the CDI and MASC with the assistance of trained psychologists. When necessary, following the manual’s standard instructions, questions were read and eventually explained to the children (25, 31). The Italian version of Child Behavior Checklist (CBCL) was used to rate children and adolescents’ behavioral and emotional problems. The CBCL is an extensively used tool that provides scores for three broad-brand scales: internalizing symptoms, externalizing symptoms and total behavioral problems. Sub items of these three broad-band scales included eight syndrome scales. Raw scores for each clinical factor were transformed into T-scores based on published norms: T-scores more than 63 were considered indicative of clinical impairment for the three broad-band scales, whereas T-scores equal or more than 70 were considered indicative of clinical impairment for Syndrome scales (33-35). The psychometric properties of the CBCL reveal good validity and reliability (36-38). The CBCL was completed by the parents of children and adolescents included in the study. The World Health Organization Quality of Life-BREF (WHOQOL-BREF) is a self-administered instrument for measuring the perception of quality of life (QOL). In the present study it was completed by the mothers of 40 NAFLD and 39 control children and adolescents to assess their perception of QOL of their sons and daughters. The WHOQOL-BREF produces a profile with four domain scores (physical, psychological, relationship and environment) and two individually scored items about an individual's overall perception of QOL (Q1) and health (Q2) (39). The WHOQOL-BREF domain scores reveals good discriminant validity and internal consistency of subscales evaluated by Cronbach’s α = 0.75. The psychometric properties of the Italian version of the WHOQOL-BREF have been tested by the “Centro Italiano Collaborativo Progetto WHOQOL” (40).

### 3.5. Data Analysis

Statistical analyses were performed by SPSS version 16.0. The data were normally distributed as shown by the Kolmogorov-Smirnov test performed for each variable used. Comparisons between NAFLD and control group were performed using Chi-square for non-parametric data and Student's *t*-tests, one-way ANOVA and linear correlations for continuous variables.

## 4. Results

### 4.1. Demographic and Clinical Characteristics

Present study includes 96 children and adolescents, 57 (39 males and 18 females, average age 12 years) were affected by non-alcoholic fatty liver disease (NAFLD) and 39 (14 males and 25 females, average age 11 years) were included in the healthy subjects group. NAFLD and healthy subjects were similar in age and distribution, as well as for the socioeconomic status of their families defined by parental education. However, in the NAFLD group there was an excess of males (73% vs. 27%, X^2^ = 8.8, P = 0.003). Table 1 demonstrates the characteristics of the study subjects.

### 4.2. Emotional and Behavioral Profile

NAFLD children scored significantly higher in the total scores of the two completed self-rating scales (MASC and CDI) as compared to healthy subjects: 49.01 ± 9.8 versus 42.9 ± 6.5 (P = 0.001) for total MASC and 7.8 ± 3.9 versus 4.3 ± 2.1 (P < 0.001) for CDI total scale, respectively. MASC subscales “Anxiety Disorder Index”, “Social Anxiety” and “Separation/Panic” also revealed higher scores for NAFLD children in comparison with healthy subjects (Table 2). The CBCL analysis, completed by the parents, revealed significantly higher scores in total problem (P = 0.046), internalizing symptoms (P < 0.001) and somatic complaints (P < 0.001) scores in the NAFLD group as compared to the control group (Table 2).

**Table 2.** Emotional and Behavioural Profile: Differences between Non-Alcoholic Fatty Liver Disease and Control Groups

Groups	NAFLD Group (n = 47)	Control Group (n = 39)	P value
**CBCL Total**	55.0 ± 9.2	51.1 ± 9.1	0.046
**CBCL Int**	59.3 ± 9.4	52.2 ± 8.4	< 0.001
**CBCL Ext**	50.7 ± 9.6	51.7 ± 8.5	NS^a^
**MASC Total**	49.0 ± 9.8	42.9 ± 6.5	0.001
**ADI**	44.2 ± 9.6	39.9 ± 6.3	0.016
**MASC Subitems**^b^			
**Physical Symptoms**	48.9 ± 7.2	47.4 ± 5.4	NS^a^
**Harm Avoidance**	43.4 ± 8.7	40.9 ± 9.2	NS^a^
**Social Anxiety**	51.9 ± 9.3	45.8 ± 6.5	0.001
**Separation/Panic**	52.9 ± 11.2	44.9 ± 6.9	< 0.001
**CDI Total**	7.8 ± 3.9	4.3 ± 2.1	< 0.001

Abbreviations: ADI, Anxiety Disorder Index; CBCL Ext, Child Behavior Checklist Externalizing Score (T-Scores); CBCL Int, Child Behavior Checklist Internalizing Score (T-Scores); CBCL Total, Child Behavior Checklist Total Score (T-Scores); CDI Total, Children Depression Inventory Total Score; MASC Total, Multidimensional Anxiety Scale for Children Total Score (T-Scores)

^a^NS Indicates P > 0.05

^b^Syndrome Scale Scores

### 4.3. Quality of Life in Parents of NAFLD and Healthy Children

The WHOQOL-BREF, completed by the mothers, showed significantly lower scores in the NAFLD group in the overall perception of quality of life (Q1) item (P < 0.001) and in the “relationships” domain (P = 0.023) when compared to the control group (Table 3).

**Table 3.** Comparison of Quality of Life (WHOQOL-BREF): Differences between Non-Alcoholic Fatty Liver Disease and Control Groups

Groups	NAFLD Group (n = 40)	Controls Group (n = 39)	P value
**Q1**^b^	65.6 ± 18.5	77.9 ± 13.5	< 0.001
**Q2**^c^	63.6 ± 24.5	71.4 ± 13.7	NS^a^
**Physical**	70.7 ± 14.2	68.4 ± 15.6	NS^a^
**Psychological**	56.9 ± 13.6	64.3 ± 15.4	0.023
**Relationship**	72.4 ± 16.5	72.2 ± 18.5	NS^a^
**Environment**	58.6 ± 15.8	54.2 ± 19.0	NS^a^

Abbreviations: NAFLD, non-alcoholic fatty liver disease

^a^NS Indicates P > 0.05

^b^Overall Perception of Quality of Life

^c^Overall Perception of Health

### 4.4. Gender Differences between NAFLD and Healthy Subjects Group

**Figure 1.**
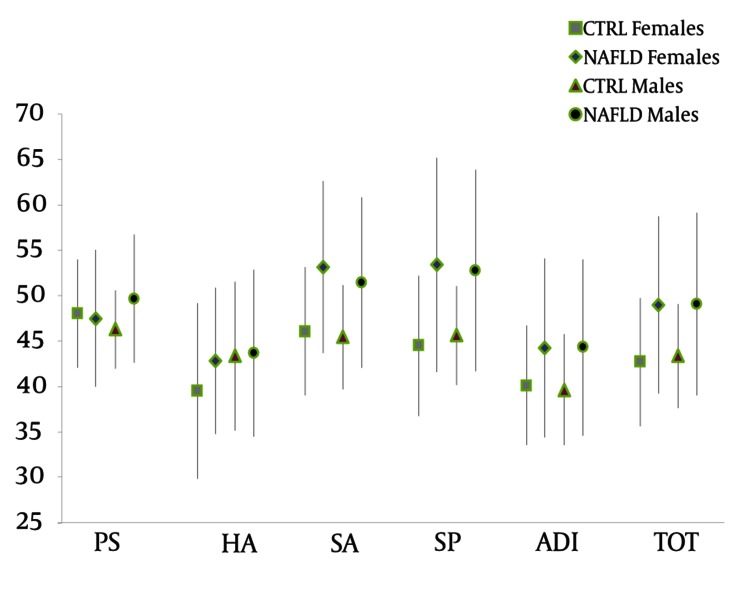
MASC Profile in Non-Alcoholic Fatty Liver Disease and Healthy Subjects: Gender Differences Abbreviations: NAFLD, non-alcoholic fatty liver disease; CTRL, controls; PS, physical symptoms; HA, harm avoidance; SA, social anxiety; SP, separation panic; ADI, anxiety disorder index; Tot, total

Analysis of the psychological profiles according to gender revealed only one significant difference within the NAFLD group whereas females scored were higher than males in the CDI subscale “Social Relation” (3.3 ± 1.9 versus 2.1 ± 1.8, P = 0.034). Within the same gender, significant differences between NAFLD and healthy subjects (NAFLD males versus healthy males and NAFLD females versus healthy females) were also found (Figure 1). In particular, NAFLD females scored significantly higher than healthy females in total MASC (49.00 ± 9.7 versus 42.6 ± 7.0, P = 0.018), MASC subscales “Social Anxiety” (53.1 ± 9.4 versus 46.0 ± 7.0, P = 0.007) and “Separation/Panic” (53.3 ± 11.7 versus 44.5 ± 7.7, P = 0.005) and CDI total scale (7.0 ± 3, 8 versus 4.3 ± 2.1, P = 0.006) (Table 4). Furthermore, CDI total score (8.1 ± 3.9 versus 4.0 ± 2.2, P = 0.001) and MASC subscales “Social Anxiety” (51.4 ± 9.3 versus 45.4 ± 5.6, P = 0.029) and “Separation/Panic” (52.7 ± 11.0 versus 45.6 ± 5.4, P = 0.026) were significantly higher in NAFLD males as compared to healthy males (Table 4). CBCL analysis also revealed higher scores in NAFLD females, as well as in NAFLD males, as compared to healthy females and healthy males, in internalizing symptoms (59.3 ± 12.0 versus 52.6 ± 7.8, P = 0.035 and 59.3 ± 8.1 versus 51.5 ± 9.5, P = 0.005, for females and males, respectively) and somatic complaints (60.6 ± 8.5 versus 52.5 ± 3.4, P = 0.000 and 59.6 ± 7.7 versus 52.4 ± 5.0, P = 0.002, for females and males, respectively) (Table 4).

**Table 4.** Gender Differences between Non-Alcoholic Fatty Liver Disease and Controls

	NAFLD Group (n = 57)	Control Group (n = 39)	P value^a^
	Females (n = 18)	Females (n = 25)	
**MASC Total, Mean ± SD**	49.00 ± 9.7	42.6 ± 7.0	0.018
**MASC Subitems, Mean ± SD**			
Social Anxiety	53.1 ± 9.4	46.0 ± 7.0	0.007
Separation/Panic	53.3 ± 11.7	44.5 ± 7.7	0.005
**CDI Total**	7.0 ± 3.8	4.3 ± 2.1	0.006
**CBCL Int**	59.3 ± 12.0	52.6 ± 7.8	0.005
**CBCL Som Comp**	60.6 ± 8.5	52.5 ± 3.4	< 0.001
**MASC Subitems, Mean ± SD**	Males (n = 39)	Males (n = 14)	
Social Anxiety	51.4 ± 9.3	45.4 ± 5.6	0.029
Separation/Panic	52.7 ± 11.0	45.6 ± 5.4	0.026
**CDI Total**	8.1 ± 3.9	4.0 ± 2.2	0.001
**CBCL Int**	59.3 ± 8.1	51.5 ± 9.5	0.005
**CBCL Som Comp**	59.6 ± 7.7	52.4 ± 5.0	0.002

Abbreviations: CBCL Int, Child Behavior Checklist Internalizing Score (T-Scores); CBCL Som, Child Behavior Checklist Somatic Complain Score (T-Scores); CDI Total, Children Depression Inventory Total Score; MASC Total, Multidimensional Anxiety Scale for Children Total Score (T-Scores); MASC Subitems, Syndrome Scale Scores; NAFLD, non-alcoholic fatty liver disease

^a^Indicates P > 0.05

### 4.5. Relationship between Psychological Profile and Biological Parameters in NAFLD Group

Linear correlation analysis was used to determine the relationship between depressive symptoms (assessed by CDI total score) and biological variables (BMI, HOMA-IR, and cytokine IL-6, cytokine TNF-α, liver enzymes-AST, liver enzymes-ALT, cholesterol total levels and triglycerides levels). No significant results were detected. Similarly, linear correlation analysis was performed to evaluate the relationship between anxiety symptoms (assessed by MASC Total score, MASC anxiety disorder index and the four MAC subscales measuring physical symptoms, social anxiety, harm avoidance and separation anxiety) and biological variables (BMI, HOMA-IR, cytokine IL-6, cytokine TNF-α, liver enzymes-AST, liver enzymes-ALT, cholesterol total levels, and triglycerides levels) also revealed no significant results. Finally, no significant correlations were detected, by linear correlation analysis, between behavioral and emotional profile (evaluated by CBCL Internalizing, CBCL Externalizing, CBCL Total Problems and CBCL eight syndrome scales) and the biological variables (BMI, HOMA-IR, cytokine IL-6, cytokine TNF-α, liver enzymes-AST, liver enzymes-ALT, cholesterol total levels, and triglycerides levels).

## 5. Discussion

Understanding the association between physical and psychological problems in young patients suffering from NAFLD is an important aspect that can provide insight on the multiple factors involved in this disease. In the present study we have evaluated the emotional and behavioral profile of children suffering from NAFLD in order to eventually identify specific psychological features. Former reports revealed a high prevalence of anxiety and depression disorders in adult patients with NAFLD (20, 41), associated with more advanced liver histological abnormalities. The hypothesis of an association with psychiatric symptoms such as low mood is also supported by other studies showing that patients with depression tend to have glucose intolerance and elevated insulin levels following oral glucose loading, and that treatment of depression and anxiety can improve hyperglycemia in diabetic individuals (42-45). Consistent with this line of reasoning, we can speculate that depression and anxiety are treatable conditions that may represent modifiable risk factors that may possibly predispose the individual to the development of NAFLD. Indeed, the results of this study, which documented increased rates of anxiety and depression symptoms in children suffering of NAFLD as compared to healthy subjects, are consistent with the former in adults (20). At a more granular level, NAFLD children revealed significantly higher scores for anxiety, social anxiety and separation panic indexes as compared to healthy controls. Furthermore, the behavioral profile discerned by CBCL revealed a higher prevalence of internalizing symptoms in NAFLD children, even following to gender matching. Finally, mothers of children with NAFLD showed a significantly worse perception of the QOL than mothers of healthy children, with significant differences in the overall perception of the QOL and in the context of relationships. In our study, the lack of association between insulin resistance measured by HOMA-IR and anxiety and depression scores, seems to exclude that these two psychiatric conditions may be related to insulin resistance, at least in our pediatric NAFLD patients. Inflammatory cytokines have also been hypothesized elsewhere to play a role in NAFLD and the same cytokines were also found to be increased in depressed patients, thus suggesting a common causative effect (46, 47). However, in our study no significant association was found with the level of inflammatory cytokines, therefore ruling out this hypothesis. The association between NAFLD and depression or anxiety could be explained by the fact that chronic illnesses can play an indirect role on subjects that have a psychological vulnerability, in terms of both development and management of the psychiatric disorder. Moreover, a different resiliency to cope with the systematic and routinely therapeutic approach for the chronic illness could also influence the emotional feeling of the patients and therefore exacerbate depressive and anxious symptoms (48). Even if anxiety and depression in our study were found more prevalent in subjects with NAFLD, further investigation is needed to better assess the association between physical and psychological problems in these patients. In addition, the present study has some important limitations that have to be taken into account. Firstly, the small sample size, in particular the control group; secondly, the absence of biological parameters in the control group for the comparison with the NAFLD children; thirdly, the lack of assessment of anxiety and depression in NAFLD mothers using specific tools other than the WHOQOL-BREF itself, a strategy that could have helped rule out the possibility of the influence of these two psychiatric symptoms in their own perception of the quality of life, and fourthly the unequal distribution of genders among the study and control group. Furthermore, the sample was entirely derived from a single clinic, and therefore it was a clinically referred sample not intended to be representative of children with NAFLD in the general population in Italy or in other clinical and academic settings. Finally, we have to point out that scores were entirely derived from self-report assessment scales completed by the parents or the children themselves and although these rating scales were shown to be a valid instrument for screening, formal diagnosis of psychiatric disorders cannot be inferred based solely on them. Indeed, given the parents’ reports, higher scores of emotionality were found in both NAFLD children and their parents, therefore we cannot completely exclude that the emotionality of the informants has somehow affected the children’s ratings, thus resulting in an overestimation of emotional and behavioral symptoms. Despite these limitations, the results presented herein, seem to indicate a higher prevalence of psychological problems in children suffering from NAFLD, which suggests that these patients may benefit from a psychological support. Ideally, this type of psychological support should also involve parents, whose quality of life seems to be negatively affected by the occurrence of this disease in their children, as well as school teachers, who may play an essential role in promoting social adaptation. In a former study from our group we presented that a cognitive-behavioral therapy might be useful for improving emotional and behavioral disorders in children with a chronic and painful diseases. Based on these findings, we conclude that psychological support at a minimum would likely be a needed and beneficial to augment treatment of NAFLD patients (49).
